# Effects of agricultural practices on foraging habitats of a seabird species in the Baltic Sea

**DOI:** 10.1002/ece3.9551

**Published:** 2022-11-22

**Authors:** Stefan Garthe, Philipp Schwemmer, Ulrike Kubetzki, Bernd Heinze

**Affiliations:** ^1^ Research and Technology Centre (FTZ) Kiel University Büsum Germany; ^2^ Verein Langenwerder zum Schutz der Wat‐ und Wasservögel e.V. Nossendorf Germany

**Keywords:** agriculture, foraging, GPS telemetry, habitat selection, seabird

## Abstract

Omnivorous and opportunistic species may be good indicators of food availability. Gulls often use human‐impacted landscapes and may respond to changes by altering their feeding ecology. We investigated the foraging behavior of individual common gulls (*Larus canus*), focusing on their distribution during foraging and their selected habitat types. We tracked adult common gulls using GPS telemetry at their largest breeding colony in the southwestern Baltic Sea, Germany. Foraging habitats were analyzed from tracking data for three breeding seasons 2016, 2017, and 2019 and were compared with potentially available foraging habitats. Most breeding birds flew toward terrestrial areas. Feeding sites were located on average 11.7–14.3 km from the colony (range 0.9–36.5 km). Corn and sugar beet fields were used significantly and extensively compared with their availability in 2016 and 2017, while wheat, rape, and barley fields were used significantly less. Data from 2019 suggested seasonal shifts in habitat use. Birds spent between 30 and 1300 min per week at their preferred feeding sites, with significant differences between the major habitats selected. We found a stable, clear, multiyear pattern in common gull foraging behavior in relation to agricultural practices. Fields with little or no crop cover and thus access to the soil were preferred over fields with high crop cover. These results suggest that local food availability may be limiting further population increases in this species.

## Graphical abstract

Most breeding Common Gulls (*Larus canus*) at their largest breeding colony in the southwestern Baltic Sea flew toward terrestrial areas for foraging. Fields with little or no crop cover and thus access to the soil were preferred over fields with high crop cover. These results suggest that local food availability may be limiting further population increases in this species.

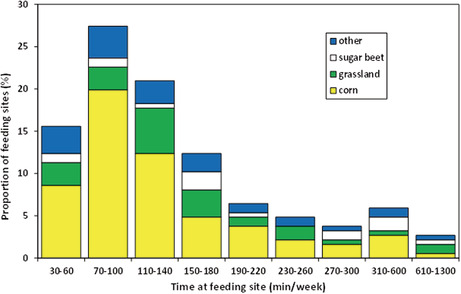

## 1 INTRODUCTION

Omnivorous and opportunistic species may be good indicators of the availability of food and its changes over time, because of their wide range of responses (Montevecchi, 1993). Gulls, as a taxonomic group, include various species known to show such behavior; they use habitats ranging from the open sea to inland areas for foraging and also respond strongly to human activities (Coulson, 2019). Their responses include the use of discards and offal from fishing vessels (Camphuysen, 1995; Sommerfeld et al., 2016), the exploitation of landfill or waste management facilities (Belant et al., 1998; Fuirst et al., 2018; Gentes et al., 2015; Horton et al., 1983; Shaffer et al., 2017) and of fishery or meat‐processing plants or markets (Yoda et al., 2012), breeding on man‐made buildings and urban areas (Kubetzki & Garthe, 2007; Rock, 2005; Spelt et al., 2019), as well as using agricultural areas (Isaksson et al., 2016; Schwemmer et al., 2008). Human‐impacted habitats undergo frequent changes in terms of both quantity and quality as foraging sites for gulls. Studying opportunistic species may thus inform us of anthropogenic changes in land use. For example, tracking of yellow‐legged gulls (*Larus michahellis*) detected illegal activities at an officially closed landfill site (Navarro et al., 2016). Lesser black‐backed gulls (*Larus fuscus*) breeding in the Wadden Sea (North Sea) have changed their foraging targets from primarily marine to predominantly terrestrial (Corman et al., 2016; Garthe et al., 2016). While reproductive success is thought to be higher among gulls using marine resources (O'Hanlon et al., 2017; Sotillo, Baert, Müller, Stienen, Soares, & Lens, 2019), there are recent examples reporting higher breeding success for gull populations heavily using anthropogenic resources (Gyimesi et al., 2016; van Donk et al., 2017).

**FIGURE 1:**
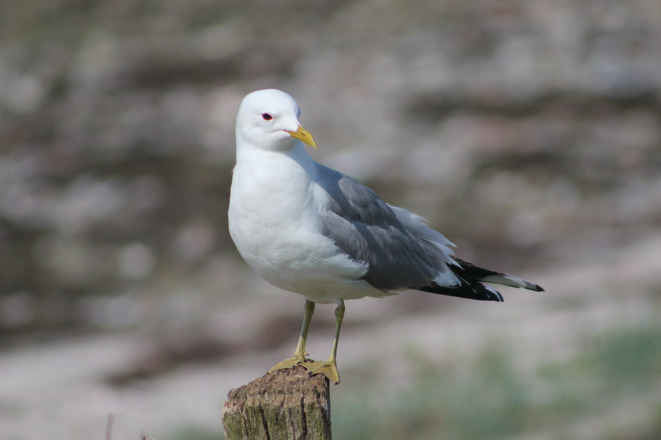
Adult common gull in the breeding colony of Langenwerder, Baltic Sea, Germany.

Based on a study of western gulls (*Larus occidentalis*) in California, Shaffer et al. (2017) concluded that population‐level plasticity may be a key factor allowing gulls to adapt to changing conditions. The common gull (*Larus canus*; Figure 1) is an opportunistic species that inhabits a wide variety of habitats and targets a wide range of prey items, ranging from mostly marine to exclusively terrestrial (Kubetzki et al., 1999; Vernon, 1972). They feed primarily on invertebrate and vertebrate animals while herbal parts of the food are mostly comprised of fruits (Glutz von Blotzheim & Bauer, 1982). Although this species is able to respond to human alterations in the seascape and landscape, common gulls have shown a long‐term population decline in all major colonies along the southwestern coast of the Baltic Sea. These declines appear to have been caused by a combination of reduced human‐produced food, changes in foraging habitats, and predation by native and non‐native predators (Kubetzki, 2001). However, although the population trends have been well documented, the importance of different foraging habitats has not yet been quantified, especially at coastal sites where foraging birds are frequently out of sight of observers. Therefore, we applied state‐of‐the‐art GPS data loggers to investigate the foraging behavior of individual common gulls at their largest colony along the southwestern Baltic Sea coast, at Langenwerder Island. We specifically addressed the following questions in this paper and describe the expected results based on former knowledge about gull foraging ecology as reported by the publications cited in this Introduction:

1. Where do common gulls forage? We expect common gulls to fly toward all directions, marine, coastal, and terrestrial, with a slight preference toward terrestrial areas as most dietary analyses showed higher percentages of food from terrestrial sources.
2. What are their preferred foraging habitat types? We expect gulls to feed in marine, coastal, and terrestrial habitats. Based on dietary analyses, preferred foraging habitats may be agricultural areas, grassland, the intertidal zone, and shallow water areas of the Baltic Sea.
3. Are there interannual differences in foraging patterns? We expect only minor differences in year‐to‐year variation because most changes in gull foraging ecology occur over longer periods (decades) as an adaption to changes in terrestrial and marine habitats and food availability.

## 2 MATERIALS AND METHODS

### 2.1 Study area

This study took place in the northwestern part of the province of Mecklenburg‐Vorpommern, located near the Baltic Sea coast in northern Germany. The study area included one large town (Rostock) surrounded by several small towns and villages embedded within an agro‐forested matrix that also included some nature protection areas along some parts of the coast. The most important crops grown in the study area based on relative cover comprised wheat, grassland, rape, corn, and barley. Grassland consisted of pastures and hayfields, and the latter were harvested throughout the entire breeding period of common gulls. No landfills or wastewater treatment plants were found in our study area.

**FIGURE 2:**
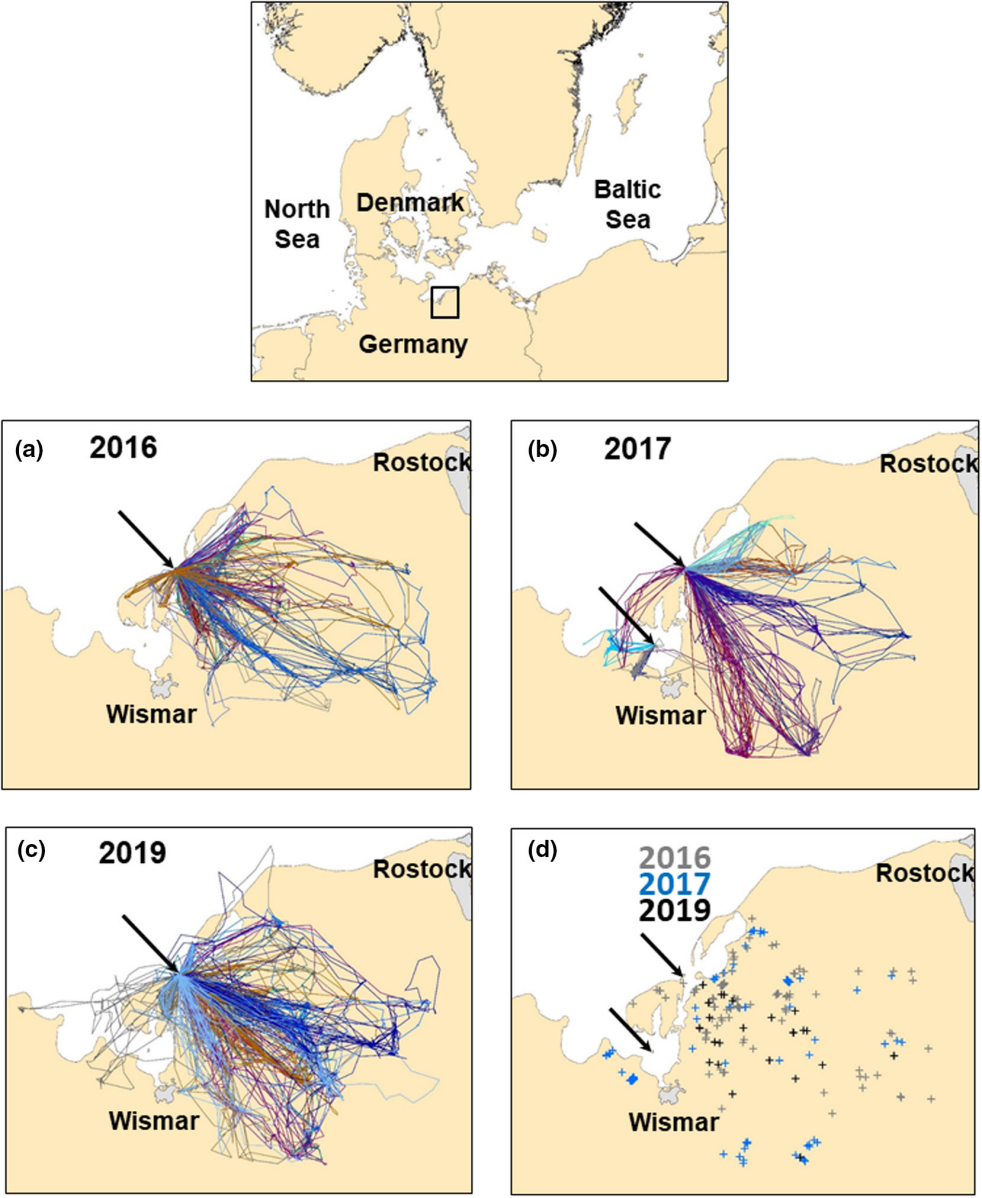
Top: Location of the study area in north‐central Europe. Small box (top center) indicates the study area in the Bay of Wismar in the Baltic Sea and represents the sector shown in the maps in this figure and Figure 3. (a–c) Flight patterns of adult common gulls from the breeding colony on Langenwerder and from the satellite colony at Walfisch (only in 2017). (a) May 14–27, 2016, *n* = 9 birds; (b) May 27–June 3, 2017, *n* = 13 birds (10 from Langenwerder, 3 from Walfisch); (c) May 25–June 14, 2019, *n* = 9 birds. The flight paths of each individual are indicated by a different color in each year. Black arrows indicate the locations of the colonies at Langenwerder and Walfisch (only in b). Data represent mostly the incubation period. (d) Locations of the most‐frequented feeding sites in 2016, 2017, and 2019 (only the first 3 weeks to match the same season as in 2016 and 2017).

We carried out fieldwork on common gulls in two breeding colonies, Langenwerder Island (54.027° N, 11.493° E), a small island in the Bay of Wismar, in the western Baltic Sea (Figure 2) primarily in 2016, 2017, and 2019 (breeding population ca. 2000 pairs) and at a satellite colony on Walfisch Island (53.940° N, 11.427° E), 10.5 km southwest of Langenwerder Island in 2017 (breeding population ca. 30 pairs).

### 2.2 Population development

The common gull population on Langenwerder has shown remarkable changes in the second half of the 20th century (Brenning & Nehls, 2013), most notably increasing until 1972, followed by the subsequent so‐called population regulation scheme (Nehls, 1974, 1979) when about 21,000 adult common gulls were killed from 1971 to 1975 and about a further 4200 adults in 1984 (see Herrmann, 2009 for a review). The population failed to recover after the second cull and the population almost halved, without any further direct human intervention. However, the local population has stabilized at a comparatively low level of around 2000 pairs since about 2003. The breeding success in recent years is apparently high enough to support the current population size, but only after establishing an anti‐predator fence around the island (Heinze & Köhler, 2014; Verein Langenwerder unpubl. data).

### 2.3 GPS data loggers

We fitted lightweight GPS data loggers (Bird Solar 10 g; e‐obs GmbH, Munich, Germany) to common gulls. The devices weighed approximately 10 g, with an additional 1.5 g of attachment material, representing an average of 2.8% of the body mass of an adult common gull (range 2.0%–3.4%; *n* = 27 individuals, mean body mass 407 g, range 337–574 g). The devices included a solar panel to recharge the battery, allowing for long‐term deployment. Data were downloaded at regular intervals from birds at the breeding colony to the e‐obs base station via a UHF link. The primary setting recorded GPS positions at 10‐min intervals (76.9% of successful GPS fixes, all data from 2016), with 20‐min intervals (22.8% of successful GPS fixes) applied when the battery power was low. Only 0.3% of the intervals between GPS fixes were 30 min or longer. None of the data sets had to be truncated due to missing GPS fixes.

### 2.4 Device attachment and duration of tracking

Nine birds were caught in 2016 using walk‐in traps, and GPS data loggers were attached to the base of the four central tail feathers using Tesa® tape (Garthe et al., 2016). Data were retrieved until the device fell off as a result of molting, and/or birds pulling off feathers, and/or departure of the birds from the colony (Table 1). From 2017 onwards, birds were tagged with the same devices, but attached using a Teflon harness backpack system (Borrmann et al., 2019). Devices were left on the birds until they either stopped working or until the birds ripped off the harness. From May 25, 2017, onwards, the tagged birds were also color‐banded to facilitate resightings. A breast feather was sampled for molecular sexing in the laboratory (Tauros Diagnostics, Berlin, Germany).

**TABLE 1:** Summary of the experiments. A: Dates of fieldwork, B: sample sizes of common gulls.

	GPS logger attachment Langenwerder	GPS logger attachment Walfisch	Feeding habitat analysis
*A: Date of fieldwork*			
2016	14 May	–	14–27 May
2017	20 + 28 May	25 May	27 May–3 June
2018	13 May	–	–
2019	25 May	–	25 May–28 June

	Langenwerder			Walfisch			Total		
	Male	Female	Total	Male	Female	Total	Male	Female	Total
*B: Sample sizes of common gulls*									
2016	6	3	9	0	0	0	6	3	9
2017	8	2	10	0	3	3	8	5	13
2019	6	3	9	0	0	0	6	3	9

### 2.5 Instrumentation effects

The aim of our GPS logger deployments was to keep the mass of the data loggers below the 3% body weight threshold, as suggested by Phillips et al. (2003). All birds with devices attached using either Tesa tape or a Teflon harness behaved apparently normally. A few individuals close to the station on Langenwerder Island were tagged each year to allow continuous and close‐distance monitoring by the wardens. They were observed to perform foraging trips regularly and were also seen to raise their chicks successfully, with no apparent difference to nontagged birds, though no quantitative data were collected yet. Possible long‐term effects were evaluated based on the return rate of birds equipped with loggers attached by harnesses in the next breeding season. In 2018, eight of 10 birds from the breeding season in 2017 could be identified (of which three were only seen while the devices were detached or no longer active). In 2019, seven of 10 birds from the breeding season in 2018 were seen (of which one was only seen while the device was detached or no longer active). It is likely that more birds without working devices were present in the colony, because not all breeding birds could be checked in detail due to the large size of the colony (>2000 pairs). However, the average resighting rate (80% from 2017 to 2018, 70% from 2018 to 2019, see above) was within the range or only slightly lower than adult survival rates reported for common gulls (Pedersen et al., 2000; Rattiste & Lilleleht, 1995). We were therefore confident that our manipulations did not have any major effect on the tagged gulls.

### 2.6 Feeding site analysis

**FIGURE 3:**
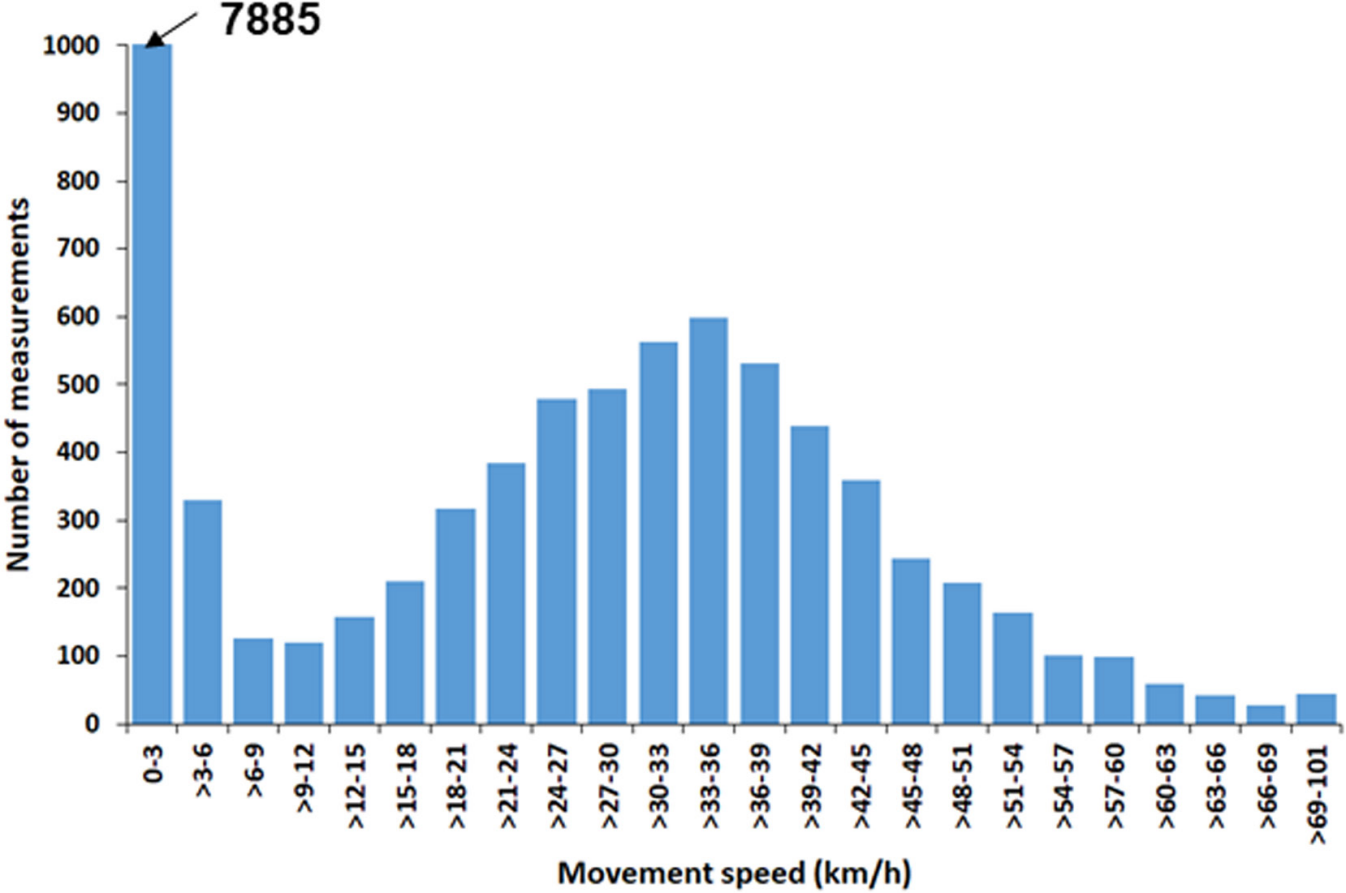
Frequency distribution of movement speeds (in km/h) of all nine common gulls recorded during their foraging trips from Langenwerder in 2016.

Feeding locations were analyzed based on the geographic positions of the GPS data from the tagged birds, following the methodology developed by Garthe et al. (2016) for lesser black‐backed gulls. Due to the fact that no spatially explicit data on habitat types (e.g., corn fields and barley fields) were available, we had to run our own sampling protocol within the same vegetation periods (i.e., before crops were harvested and identification of crop type was impossible). The use of terrestrial habitats was analyzed based on GPS positions recorded in 2016, 2017, and 2019 (details below), excluding the islands where the birds were breeding. For this, all positions until 1.0 km from the breeding colony were removed to exclude birds attending the colony. Movement speeds >10 km/h were removed from the analysis to exclude commuting flights from/to the breeding colony as well as flight movements among feeding sites (Figure 3; see also Garthe et al., 2016). Feeding sites were identified for all individuals and trips, based on the criteria that a bird spent at least 30 min in an area of a maximum size of 500 m × 500 m, following Garthe et al. (2016). This procedure was designed to identify the most important feeding sites assuming that gulls maximize their time at a site when feeding is profitable and leaving a site when feeding is not profitable. Visual observations of foraging gulls suggested that birds spending extended times in a specific location (i.e., field) were also experiencing high feeding rates (i.e., pecking or grabbing food items). If the feeding location was >500 m × 500 m (which was rarely the case), either more than one feeding site was defined, based on the above criteria, or the most intensively used area was selected. Most feeding sites were much smaller than 500 m × 500 m.

The whole procedure was carried out by analyzing the data for the 3 years. For 2016, the six most intensely used feeding sites per individual per week for 2 consecutive weeks (14–20 May and 21–27 May) were analyzed, resulting in 108 feeding sites for the nine tagged birds from Langenwerder. For 2017, the six most intensively used sites per individual for 1 week (27 May–2 June or 28 May–3 June, depending on tagging day) were analyzed, resulting in 78 feeding sites for the 10 tagged birds from Langenwerder and the three from Walfisch. In both years, 2016 and 2017, this analysis represented the incubation period of the common gulls. Hatching usually started in the last few days of May and peaked toward mid‐June. Additionally, in 2019, the single most intensively used site per individual for 5 consecutive weeks (25–31 May until 22–28 June) was analyzed, resulting in 45 feeding sites for the nine tagged birds from Langenwerder (one bird had a malfunctioning device and was thus excluded). This was done to check for possible seasonal changes. All feeding sites were visited within the same breeding season to identify the habitat type.

In 2016 and 2017, habitat type availability was mapped in parallel and on the same days that the feeding locations were visited. Transect counts of land use were carried out from a moving car throughout the whole distribution area of common gulls performing foraging flights from Langenwerder, following Garthe et al. (2016). Habitats outside towns, villages, and forests were quantified by identifying the vegetation/crop on both sides of the road every 0.5 km. This distance was chosen to avoid biases due to field units of unequal size. Each identification was taken as one unit of land use. Totals of 460 (in 2016) and 366 (in 2017) land‐use units were retained for final analysis. Habitat availability and use by common gulls were compared by Monte Carlo permutation tests, based on *χ* ^2^ tests with simulated *p* values based on 10,000 replicates per analysis. The availability and use of habitats by gulls were tested separately for each crop type.

### 2.7 Statistical analysis

We used two sets of linear mixed effect models (LME; Bolker et al., 2008; Faraway, 2006) to further analyze the feeding site behavior of the common gulls. For all LMEs, the individual bird identification number was used as random factor to account for pseudo‐replication caused by multiple observations of flight tracks by the same individual. Model selection was based on maximum‐likelihood ratio tests. Models were checked for unequal variance structures (heteroscedasticity) by plotting standardized residuals against fitted values. We inspected the qq‐plots of the residuals and the random effects to check for normality of errors. No transformation of the response variable was necessary.

In LME set (1), we analyzed in separate model runs the distances of the selected feeding sites from both the colony and the nearest coastline. We tested whether these distances were related to habitat type, with the study year as a covariate in the model. We only included habitat types that were selected by common gulls ≥10 times in total, to ensure an adequate sample size, and consequently, only differences between the four most important foraging habitats were analyzed (i.e., farm, pasture, corn, and sugar beet).

In LME set (2), we investigated the time that birds spent on feeding sites. For each feeding site, the number of GPS fixes per individual and week was summed. Since most of the fixes were obtained at the standard 10‐min interval, the number of fixes was translated into time at feeding site (with one fix equaling 10 min). It was tested whether the time at the feeding site was related to habitat type. Again, we only included habitat types that were selected by common gulls ≥10 times in total, to ensure an adequate sample size. We hence compared the time spent on foraging sites among the three field/crop types mostly used by gulls (i.e., corn, grassland, and sugar beet). Only the years 2016 and 2017 were used for this analysis because the feeding site selection in 2019 comprised much less sites and was longer in the year and was thus considered less comparable.

All statistical tests were carried out using R 3.5.1 (R Development Core Team, 2018).

## 3 RESULTS

### 3.1 Foraging flights

Most flight activities of common gulls were directed toward terrestrial areas, with very few flights toward the open sea (Figure 2a–c). This pattern was consistent across all individuals tagged. Among all GPS fixes recorded away from the colony, 95.8% of fixes in 2016, 96.2% in 2017, and 92.5% in 2019 were on land, with the remaining fixes originating from marine areas (total sample sizes for the three periods covered in Figure 1: 4009 fixes in 2016, 3207 fixes in 2017, and 10,046 fixes in 2019). The maps only cover parts of the whole data set but were primarily drawn to match the time of feeding location analysis (see below). Although there was some individual variability in foraging patterns and destinations, the overall foraging areas were quite similar across the 3 years.

### 3.2 Feeding sites

The most‐frequented feeding habitats were in fields containing corn, grassland, and sugar beet (Table 2). Differences between 2016 and 2017 and between sexes were minor (Table 2; but note the relatively small sample size for possible sex comparisons).

**TABLE 2:** Proportional use of habitat types as feeding sites by common gulls during the incubation periods in 2016 (14–27 May, *n* = 9 birds) and 2017 (27 May–3 June, *n* = 13 birds). The sample sizes were 108 sites (72 by males and 36 by females) in 2016 and 78 (48 by males and 30 by females) in 2017. Data for males and females are shown only as means of both years.

Year	2016	2017	Mean of both years	Mean of both years	Mean of both years
Sex	All	All	All	Male	Female
Habitat type					
No or little vegetation					
Corn	58.3	53.8	56.1	55.9	55.3
Sugar beet	6.5	11.5	9.0	9.7	8.6
Potato	5.6	1.3	3.4	2.4	5.6
Strawberry	0	6.4	3.2	0.0	8.3
Small Christmas trees	5.6	0	2.8	2.1	4.2
Cabbage	0.9	0	0.5	0.7	0.0
High crop cover					
Grassland	15.7	23.1	19.4	22.6	13.9
Farm/silage factory	3.7	2.6	3.1	4.2	1.4
Wheat	3.7	0	1.9	1.4	2.8
Fallow	0	1.3	0.6	1.0	0.0

**FIGURE 4:**
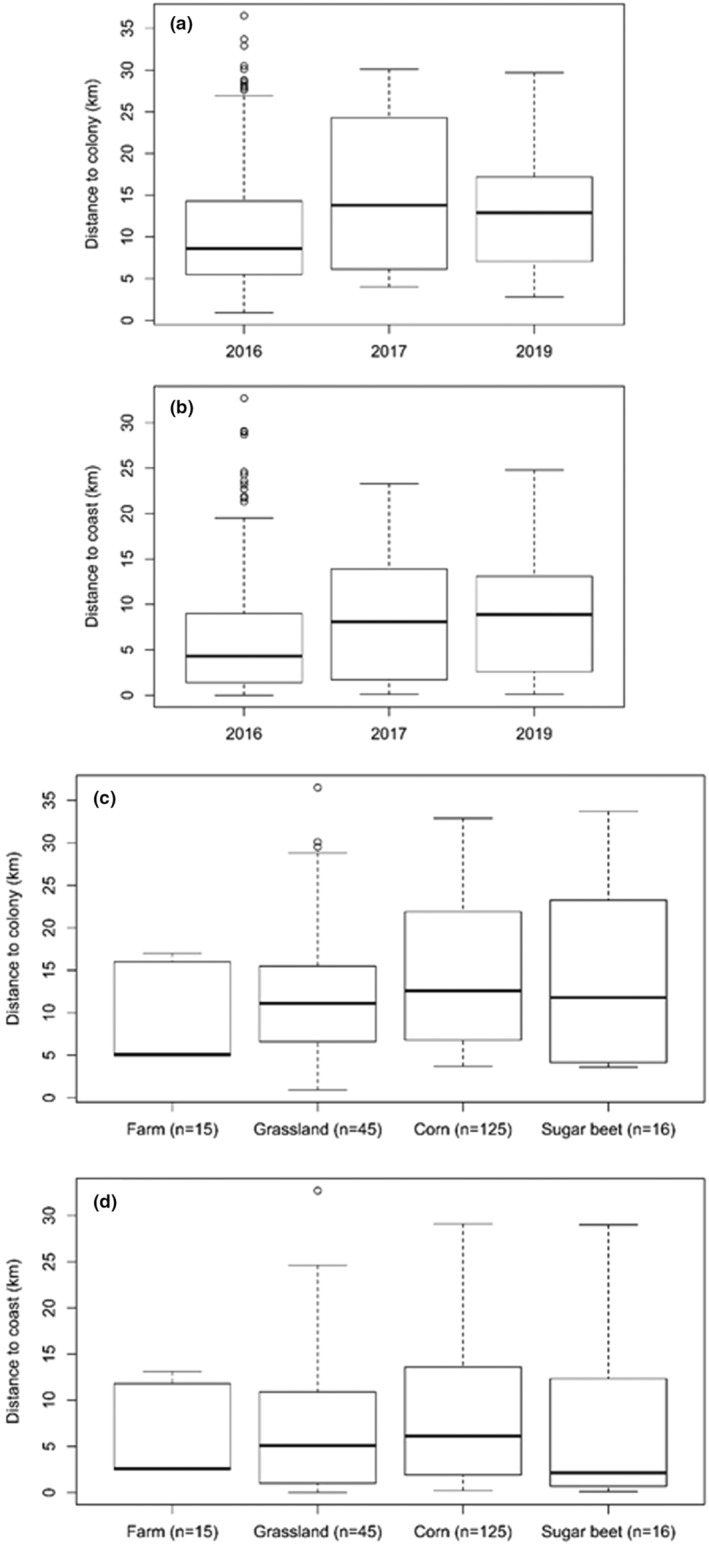
(a, b) Differences between years in the distances of the most‐frequented feeding sites of common gulls from (a) the colony and (b) the nearest coastline. Please note that only feeding sites of the major habitat types were included (same sample size as in c and d). (c, d) Differences between habitat types in the distances of the most‐frequented feeding sites of common gulls from (c) the colony and (d) the nearest coastline. Box: 50% of the data between the first (25%) and third (75%) quartile; whiskers: 25% of the remaining data; horizontal black line: median; dots: outliers.

Most‐frequented feeding sites were mostly located to the east and southeast of Langenwerder (Figure 2d). Many sites were situated relatively close to the colony and coastline, but several feeding sites were further away. The average distances of the feeding sites from the colony were 11.7 km in 2016 (range 0.9–36.5 km, *n* = 108), 14.3 km in 2017 (range 4.0–30.1 km, *n* = 78), and 13.6 km in 2019 (range 2.8–29.7 km, *n* = 45). The distances of the most‐frequented feeding sites from the colony differed significantly between years (*χ* ^2^ = 9.14, df = 2, *p* = .01, LME) but not the distances of the feeding sites from the nearest coastline (*χ* ^2^ = 2.5, df = 2, *p* = .29; Figure 4a,b). However, there were no statistical differences in the distances of the four main habitat types used as feeding sites when accounting for variability among years; distance to colony: *χ* ^2^ = 1.99, df = 3, *p* = .575; distance to coast: *χ* ^2^ = 0.73, df = 3, *p* = .865 (LME; Figure 4c,d).

**FIGURE 5:**
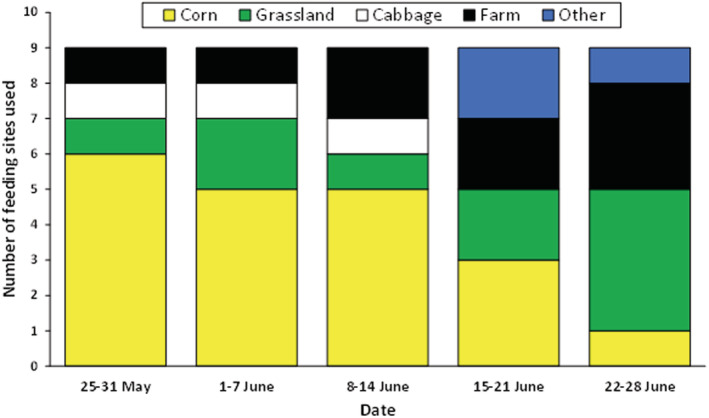
Number of feeding sites used by common gulls over the breeding period in 2019 (*n* = 9 birds).

An additional analysis from 2019 suggested the existence of seasonal shifts in the habitats used for feeding, with a decreasing importance of corn fields over the breeding season and increasing importance of other habitats (Figure 5).

The habitats used for feeding were not used in proportion to their availability (Table 3). Corn and sugar beet fields were used significantly and extensively in both years, and strawberry fields, potato fields, and grassland were used extensively in one of the 2 years. By contrast, wheat, rape, and barley fields were used significantly less in proportion to their availabilities in both years.

**TABLE 3:** Availability of habitat types and their uses as feeding sites by common gulls tagged in the Bay of Wismar in 2016 (14–27 May, *n* = 9 birds) and 2017 (27 May–3 June, *n* = 13 birds). Data represent the incubation period of the common gulls. Differences between available and used habitats were tested using Monte Carlo permutation tests.

Habitat type	2016				2017			
	Availability(% of sites)	Feeding sites (%)	*χ* ^2^	*p*	Availability (% of sites)	Feeding sites (%)	*χ* ^2^	*p*
Total sites	460	97			366	76		
*No or little vegetation*								
Corn	13.9	64.9	189.080	*<.0001*	14.5	55.3	102.070	*<.0001*
Potato	1.5	6.2	12.520	*.005*	0.8	1.3	0.230	1.000
Sugar beet	0.4	7.2	95.232	*<.0001*	0.8	11.8	113.580	*<.0001*
Strawberry	0	0		–	0.3	6.6	110.91	*<.0001*
*High crop cover*								
Wheat	27.0	4.1	28.210	**<.0001**	33.1	0	37.535	**<.0001**
Rape	20.0	0	26.000	**<.0001**	16.4	0	14.902	**<.001**
Grassland	17.4	17.5	0.079	.796	13.9	23.7	6.024	*.020*
Barley	12.6	0	15.005	**<.0001**	13.4	0	11.748	**<.001**
Rye	2.0	0	2.075	.187	4.6	0	3.702	.054
Mixed herbs	1.3	0	1.374	.412	0.3	0	0.208	1.000
Pea	1.3	0	1.374	.412	0.3	0	0.208	1.000
Oat	1.1	0	1.143	.430	0	0		–
Fallow	0.7	0	0.683	.657	0.3	1.3	3.032	.183
Broad bean	0.4	0	0.454	1.000	1.1	0	0.840	.631
Clover	0	0		–	0.3	0	0.208	1.000

*Note*: Significant results in italic relate to habitats used more than available on average, and results in bold relate to habitats used less than available on average. Please note that farm/silage factory and small Christmas trees were excluded from this analysis as they could not be quantified in the same way.

**FIGURE 6:**
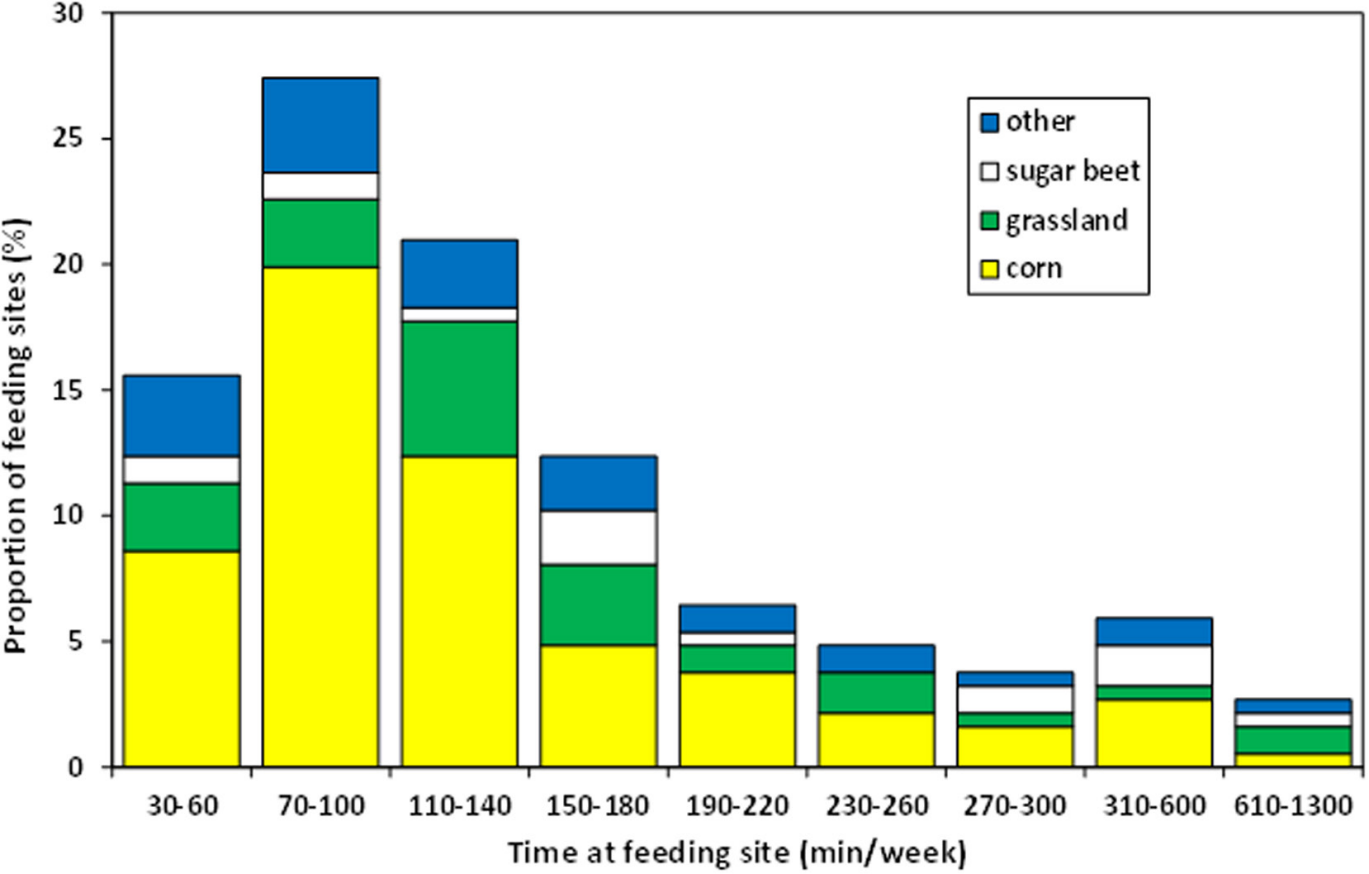
Estimated time spent by common gulls at their preferred feeding sites, separated for different habitat types. Data originate from the incubation periods in 2016 (*n* = 9 birds) and 2017 (*n* = 10 birds at Langenwerder and 3 birds at Walfisch).

Birds spent between 30 and 1300 min per week at their preferred feeding sites (Figure 6). The single longest, continuous visit was 500 min. The time spent at the feeding site differed significantly between the three habitats (LME: *χ* ^2^ = 7.5, df = 2; *p* = .023). On average, common gulls stayed longer at their feeding sites on sugar beet fields (mean = 254 min, range 60–1020 min, *n* = 16) than at grassland (mean = 190 min, range 40–1300 min, *n* = 35) and corn fields (mean = 137 min, range 30–650 min, *n* = 105).

## 4 DISCUSSION

### 4.1 Feeding habitats

Common gulls tagged in the Bay of Wismar used almost exclusively terrestrial habitats, with most feeding sites located in agricultural areas. This pattern initially appears to follow recent findings in other *Larus* species, for which a switch from marine to terrestrial habitats has been reported at various locations during the last few years (Enners et al., 2017; Garthe et al., 2016; Gyimesi et al., 2016; Sotillo, Baert, Müller, Stienen, & Lens, 2019). However, terrestrial diets in common gulls were also reported in a very early study by Wachs (1939), who found that the predominant food items of common gulls were mice and insects caught in agricultural areas. Terrestrial food sources were also identified as the main dietary components of common gulls along the Baltic Sea coast in the German federal state of Schleswig‐Holstein several decades later (Kubetzki, 2001; Kubetzki & Garthe, 2007), while the diet along the North Sea coast was much more marine (Kubetzki et al., 1999). Although the food consumed by gulls from Langenwerder in this tagging study has not yet been analyzed in detail, pellets collected contained almost exclusively terrestrial food items, including high proportions of earthworms and insects, thus confirming our findings regarding the spatial foraging patterns. Individual patterns of habitat use by larger *Larus* species have been tracked using GPS tags (Enners et al., 2017; Fuirst et al., 2018; Garthe et al., 2016; Gyimesi et al., 2016; O'Hanlon et al., 2017; Shaffer et al., 2017; Sotillo, Baert, Müller, Stienen, & Lens, 2019); however, similar information for the smaller common gulls is lacking, except for data from three tagged individuals in Sweden in 2014 (Evans, 2017). Progress in the miniaturization of GPS tags allowed us to conduct the current study, which succeeded in creating the first comprehensive data set regarding the habitat use of individual common gulls. The findings of this study clearly support a terrestrial foraging habitat choice by common gulls in the Baltic Sea region, as previously reported in studies of diet remains (Kubetzki, 2001; Kubetzki & Garthe, 2007). While no dedicated analysis on individual specialization on the choice of foraging sites in common gulls has been made, Figure 2 and unpubl. data suggest this to occur. Individual specialization has implications for life histories and population dynamics (Annett & Pierotti, 1999; Phillips et al., 2017). Borrmann et al. (2019) found clear spatial segregation between individual great black‐backed gulls (*Larus marinus*) in the Wadden Sea, with almost no overlap of their core areas. Along the southern North Sea coast, lesser black‐backed gulls specializing on discard utilization appeared to be able to flexibly respond to the temporary loss of discards by switching to alternative resources (Tyson et al., 2015). Possibly representative for various gull species and populations could be results from a detailed study on yellow‐legged gulls *Larus michahellis* in the Mediterranean Sea: Navarro et al. (2017) revealed that population‐level generalism in habitat use arises through varying levels of individual specialization and individual spatial segregation within each habitat.

Closer analysis of the utilization of the terrestrial habitat mosaic by common gulls revealed a clear preference for corn and sugar beet fields in terms of feeding site selection during the incubation period in late May in both 2016 and 2017. This might seem unexpected, but notably, fields sown with these crops have very little vegetation at that time, giving the birds direct access to the ground and allowing them to feed on arthropods and earthworms, which were reported to comprise a major proportion of the diet of common gulls in previous studies (Kubetzki, 2001; Kubetzki & Garthe, 2007; Vernon, 1970, 1972). A similar situation was shown for potato and strawberry fields, both of which were used extensively by the gulls in 1 year. This finding is similar to that in a study of lesser black‐backed gulls along the German North Sea coast, where gulls preferentially foraged on bare ground, with significantly higher use of potato fields and significantly less use of grassland (Garthe et al., 2016). Similarly, numbers of ring‐billed gulls (*Larus delawarensis*) were higher in fields covered by bare soil and peaked during soil preparation and seed sowing, which greatly increased food availability (Patenaude‐Monette et al., 2014; Schwemmer et al., 2008). Isaksson et al. (2016) showed that lesser black‐backed gulls along the Baltic Sea coast in Sweden foraged preferentially in agricultural areas with short vegetation, with decreasing intensity later in the breeding season.

### 4.2 Long‐term changes in foraging habitats

Selection of feeding habitats during the incubation period was apparently not influenced by the distance to the colony or the nearest coastline. The importance of root crops and corn increased when the cultivation of wheat and barley changed from predominantly summer to winter crops in the 20th century (for details, see Backhaus, 2001). Because corn and sugar beet grow during June and July, access to the soil by the gulls becomes increasingly restricted. The relative importance of corn fields should decrease while that of other habitats should increase, as confirmed by the first data collected in 2019. It is known that gulls move to other spots when feeding efficiency is low (e.g., shown for gulls feeding on fishery waste; Camphuysen et al., 1995). From the time spent foraging at a feeding site, it might thus be concluded that sugar beet fields are the most preferred feeding habitat.

Grassland has been described as the most important feeding habitat for common gulls near the German North Sea coast (Schwemmer et al., 2008, 2017); however, this habitat was overproportionally used in our study only in 2017. There are several possible explanations for this observation. Although there has been no overall decrease in grassland over the last 20 years in the province of Mecklenburg‐Vorpommern where the study area is located (1999: 2823 km^2^, 2009: 2685 km^2^, 2019: 2696 km^2^; reports of the Statistisches Amt Mecklenburg‐Vorpommern), the quality of the grassland has apparently deteriorated. Formerly wet grasslands have become dry, and they are thus only a major attraction for foraging common gulls when precipitation is high, as in spring 2017, and many grassland areas were covered by water. Furthermore, grassland has been subject to intensified management practices, including regular mowing and low numbers of grazing cattle. This might explain the lower attraction to common gulls, given that previous studies found a significantly reduced abundance of arthropods in mowed grassland compared with grazed sites (Barnett et al., 2004; Vickery et al., 2001).

The use of corn has increased substantially over the last 20 years, due to new demands for biofuel (Di Lucia et al., 2012; Reise et al., 2012). Although this practice offers common gulls access to the soil, particularly during the first half of the breeding season, as shown in this paper, the increase in corn fields is controversial. The implementation of biofuels has negative implications, particularly from a nature conservation point of view, given that other habitats, especially grassland, have been converted into corn fields and thus become lost. Furthermore, corn requires extensive use of pesticides, which are detrimental to wildlife (Atwood et al., 2018; Meissle et al., 2010).

## 5 CONCLUSIONS

We found a stable, clear, multiyear pattern of foraging behaviors in common gulls, primarily in relation to agricultural practices. Fields with little or no crop cover were preferred over fields with high crop cover because ground‐based invertebrate food was much better accessible. Grassland was also important, but was less important than fields of root crops and corn, at least during the early breeding period.

Despite their wide distribution, surprisingly little is known about the foraging ecology and habitat selection of common gulls. This is particularly true of the behavior of this species independent of human activities, and there is a lack of information on common gulls in this region before anthropogenic actions affected their foraging behavior, both directly and indirectly. However, the results suggest that interspecific competition is likely to increase, given that other gull species are also currently shifting their habitats from marine to terrestrial sites.

## AUTHOR CONTRIBUTIONS

**Stefan Garthe:** Conceptualization (lead); formal analysis (lead); funding acquisition (lead); investigation (equal); methodology (lead); project administration (lead); resources (equal); visualization (equal); writing – original draft (lead). **Philipp Schwemmer:** Formal analysis (supporting); investigation (equal); methodology (supporting); resources (equal); visualization (equal); writing – review and editing (equal). **Ulrike Kubetzki:** Investigation (equal); resources (equal); writing – review and editing (equal). **Bernd Heinze:** Investigation (equal); resources (equal); writing – review and editing (equal).

## CONFLICT OF INTEREST

The authors declare that they have no conflict of interest with the content of this article.

## Data Availability

The datasets generated and analyzed during the current study are available in the Movebank Data Repository, https://doi.org/10.5441/001/1.p44ms6mr (Garthe et al., 2022). Bird‐ringing data are archived at the Beringungszentrale Hiddensee (www.beringungszentrale‐hiddensee.de).
